# Dynamic Analysis and Optimal Control of Fractional Order African Swine Fever Models with Media Coverage

**DOI:** 10.3390/ani13142252

**Published:** 2023-07-09

**Authors:** Ruiqing Shi, Yihong Zhang, Cuihong Wang

**Affiliations:** School of Mathematics and Computer Science, Shanxi Normal University, Taiyuan 030031, China; zhangyihong98@163.com (Y.Z.); sxwangcuihong@163.com (C.W.)

**Keywords:** African swine fever, fractional order, basic reproduction number, stability, optimal control

## Abstract

**Simple Summary:**

African swine fever, as an acute, contact-transmitted infection, has a very high mortality and infectivity, and there are no effective drugs that can treat the disease. Therefore, it is necessary to take biosecurity measures in time at the beginning of the disease outbreak. In this article, media coverage is introduced into the African swine fever model, and the results indicate that real-time media coverage of the African swine fever epidemic is beneficial for breeders to scientifically prevent the spread of the epidemic in a timely manner. Through the evaluation of specific prevention and control measures, we suggest that timely disinfection and sterilization measures taken by breeding personnel after receiving relevant reports of the swine fever epidemic can effectively control the spread of the disease.

**Abstract:**

African swine fever is a highly contagious virus that causes pig disease. Its onset process is short, but the mortality rate is as high as 100%. There are still no effective drugs that have been developed to treat African swine fever, and prevention and control measures are currently the best means to avoid infection in pig herds. In this paper, two fractional order mathematical models with media coverage are constructed to describe the transmission of African swine fever. The first model is a basic model with media coverage, and no control measures are considered. For this model, the reproduction number is obtained by using the next generation matrix method. Then, the sufficient conditions for the existence and stability of two equilibriums are obtained. Based on the first model, the second model is established incorporating two control measures. By using Pontryagin’s maximal principle, the optimal control solution is derived. After that, some numerical simulations are performed for the two models to verify the theoretical results. Both the qualitative analysis and numerical results indicate that timely media coverage combined with disinfection control measures is crucial to preventing the spread of disease.

## 1. Introduction

African swine fever (ASF) is an acute, hemorrhagic and severe infectious disease caused by African swine fever virus (ASFV) infecting domestic pigs and various wild pigs. It can infect pigs of all breeds and ages, and the mortality rate can be up to 100% [1,2]. ASF has been present in sub-Saharan African countries since 1921, when it was first reported in Kenya [3,4], and spread to Western Europe and Latin America around 1975. Although most of the disease was eradicated in time, it did not fundamentally solve the problem. After 2007, ASF once again spread in many parts of the world, especially in Russia and its surrounding areas. China, the world’s largest producer and consumer of pork, was also hit by ASF in 2018. For those countries that produce and export a lot of pork every year, the ravages of ASF can be devastating to the pig industry, the most typical example of which is Denmark [5]. Because of its serious economic consequences, ASF has been designated as a notifiable animal disease by the World Organization for Animal Health [6], and has attracted the attention of relevant experts.

So far, no vaccines or drugs have been found that can effectively prevent and control ASF. Biosecurity is still the best means to fight against ASFV at present and in the future. Dione et al., in 2020 used a randomized controlled trial to assess the impact of farmers’ participatory training on ASF control-related biosafety knowledge, attitude and practice in two regions of Uganda [7]. In 2022, Stoffel et al. investigated the driving factors behind the introduction of ASF into compartments and classified compartments based on the risk of the ASF introduction. They believe that ASF is increasingly an anthropogenic problem. In order to strengthen compliance with biosecurity measures, updating compartment standards and addressing knowledge gaps among compartment personnel in ASF are the most crucial [8].

According to research, the source of infection of ASF mainly includes two parts: invisible infected pigs and diseased pigs. The body fluids and tissue fluids of these two kinds of pigs contain ASFV. Healthy pigs will be infected through direct contact with infected pigs or pollutants [9]. In addition, healthy pigs will also be infected if they eat swill containing viruses or are bitten by ticks carrying ASFV [10]. Among them, ticks play an important role as vectors in the transmission of ASF. In their 2017 work, Frant et al. proposed that the sylvatic cycle composed of wild pigs and various ticks may continue to be a major issue in controlling ASF and may cause new outbreaks of this disease in new regions of the world [11]. In 2021, Kouidere et al. established a model describing the transmission of ASF between pigs and ticks, and they used Pontryagin’s maximum principle to derive the optimal strategy for reducing the number of infected pigs and ticks [12].

According to the transmission characteristics of ASF, many mathematical models have been established to study how to effectively control the spread of the disease. For example, Barongo et al. developed a stochastic model to assess the impact of the timing of different control strategies on disease-related mortality. Through numerical simulation, it was found that if biosafety measures were implemented before 14 days after the outbreak of ASF, 74% of pigs could avoid infection. They also hypothesized that if there were effective vaccinations to enhance immunity during this period, 91% of pig deaths could be prevented [13]. In 2020, Zhang et al. established a mathematical model to study the transmission mechanism and control strategy of ASF in large farms, indicating the necessity of disinfection measures and staff management measures in pig farms [14]. In 2022, Song et al. developed a mathematical model with the asymptomatic infection and transmission of other pollution sources to investigate the impact of culling on the spread of ASF [15].

There are many mathematical models being proposed to study the control effects of different control measures on ASF [3,6,12,16,17]. All of these efforts indicate that in the early stages of the epidemic, if relevant measures can be taken in a timely manner to prevent the further spread of the epidemic, it can effectively reduce the mortality rate of pigs. It can be observed that timely reporting of disease information plays a crucial role in controlling the epidemic. We are in the era of internet information sharing, and we can fully utilize this resource to quickly and effectively let people know how to prevent and control diseases. For example, through media coverage, people can learn about the source of infection, transmission route, clinical symptoms, preventive measures and other related knowledge to keep people alert. Different people will have different reactions after receiving the news about the disease outbreak, which can be divided into two situations. Some people reduce the contact rate between healthy individuals and infected individuals by means of isolation, increasing the safe distance, wearing masks, etc., so as to reduce the infection. This is called a weak negative feedback effect of information [18,19]. However, others ensure that healthy individuals are not infected by regular disinfection of their living environment and tableware, which is known as a strong negative feedback effect of information [20,21]. There is literature demonstrating that media coverage is indeed effective in reducing the spread of disease [22,23,24]. In [25,26], the authors proposed mathematical models, including nonlinear function as the transmission rate, and they studied the influence of media coverage on infectious diseases. In 2017, Zhao et al. considered an SIR epidemic model combining time delay and media coverage [27].

Differential equations are useful mathematical tools to modeling and analyzing different problems, such as in applied science, engineering, and biological systems. The relevant research shows that many aspects of these fields have a temporal memory [28,29]. As a generalization of the integer differential equation, the fractional order differential equation is a theory about the differential and integral of any order [30]. It can be used as a tool to better describe different fields with memory and genetic characteristics. However, integer order differential equations cannot explain this feature very well. Therefore, in recent years, more and more fractional order differential equations are used to describe problems in the fields of epidemiology and other fields [28,29,30,31,32]. It has been demonstrated that fractional order models are better or more suitable than integer order models [33]. Therefore, it is of practical significance to introduce fractional order differential equations into the study of ASF.

Based on the above discussion, in this article two fractional order ASF models with media coverage are established to explore the transmission and spread of African swine fever. Compared to integer order models, fractional order models have not been widely used in the practical application of epidemic models. As an extension of integer order models, fractional order models can better interpret the memory during disease transmission. Moreover, when taking control measures in practice, the workload of the staff is not fixed, so optimal control is more practical than constant control. Thus, the research approach of this article is worth exploring.

The structure of this article is arranged as follows: In Section 2, a basic model with media coverage is proposed, and then another model with control measures is constructed, together with the meanings and value sources for the parameters in the models. The main results are listed in Section 3. Summaries and discussions are presented in Section 4. In the Section 5, some prospects for future research directions on this topic are provided.

## 2. Materials and Methods

ASF is mainly transmitted through direct contact with diseased pigs and ASFV contaminants in the environment. According to [34], we find that the ASF data from China mainly including the number of infected pigs and the pollution sources in the environment. It is assumed that when farmers receive the news of the local outbreak of ASF, they will reduce the probability of infection by isolating sick pigs and feeding pigs without using swill or meal waste.

### 2.1. Models Formulation

Motivated by [14,15,16,25,26], we establish a fractional order ASFV model with media coverage, as follows:

(1) $$\left\{\begin{array}{ccc} \;D^{\alpha }S\left(t\right) & = & \Lambda -dS-\left(\beta_{0}-\frac{\beta_{1}I_{s}}{m+I_{s}}\right)S\left(I_{s}+\eta I_{a}\right)-\left(\beta_{2}-\frac{\beta_{3}V}{m+V}\right)SV,\\ \;D^{\alpha }I_{s}\left(t\right) & = & p\left[\left(\beta_{0}-\frac{\beta_{1}I_{s}}{m+I_{s}}\right)S\left(I_{s}+\eta I_{a}\right)+\left(\beta_{2}-\frac{\beta_{3}V}{m+V}\right)SV\right]-d_{1}I_{s},\\ \;D^{\alpha }I_{a}\left(t\right) & = & \left(1-p\right)\left[\left(\beta_{0}-\frac{\beta_{1}I_{s}}{m+I_{s}}\right)S\left(I_{s}+\eta I_{a}\right)+\left(\beta_{2}-\frac{\beta_{3}V}{m+V}\right)SV\right]-dI_{a},\\ \;D^{\alpha }V\left(t\right) & = & hd_{1}I_{s}+kdI_{a}-\varphi V, \end{array}\right.$$

with initial conditions

$$S\left(0\right)\geq 0,\;I_{s}\left(0\right)\geq 0,\;I_{a}\left(0\right)\geq 0,\;V\left(0\right)\geq 0.$$

Here, the factional order derivative is used in the Caputo sense for systems (1). The total population, denoted as N(t), is divided into three classes: susceptible population S(t), symptomatic infectious population $I_{s}\left(t\right)$, and asymptomatic infectious population $I_{a}\left(t\right)$. V(t) represents the density of ASFV in the environment. For this model, we will mainly consider the influence of media coverage on the spread of ASF.

In order to further study the effect of media coverage on the prevention and control of ASF, two control measures, denoted as $u_{1}\left(t\right)$ and $u_{2}\left(t\right)$, are added to the basic model (1). Then, the following model with control measures is derived.

(2) $$\left\{\begin{array}{ccc} \;D^{\alpha }S\left(t\right) & = & \Lambda -dS-\left(\beta_{0}-\frac{\beta_{1}I_{s}}{m+I_{s}}\right)S\left(I_{s}+\eta I_{a}\right)-\left(\beta_{2}-\frac{\beta_{3}V}{m+V}\right)SV,\\ \;D^{\alpha }I_{s}\left(t\right) & = & p\left[\left(\beta_{0}-\frac{\beta_{1}I_{s}}{m+I_{s}}\right)S\left(I_{s}+\eta I_{a}\right)+\left(\beta_{2}-\frac{\beta_{3}V}{m+V}\right)SV\right]-d_{1}I_{s}-u_{1}\left(t\right)I_{s},\\ \;D^{\alpha }I_{a}\left(t\right) & = & \left(1-p\right)\left[\left(\beta_{0}-\frac{\beta_{1}I_{s}}{m+I_{s}}\right)S\left(I_{s}+\eta I_{a}\right)+\left(\beta_{2}-\frac{\beta_{3}V}{m+V}\right)SV\right]-dI_{a},\\ \;D^{\alpha }V\left(t\right) & = & hd_{1}I_{s}+kdI_{a}-\varphi V-u_{2}\left(t\right)V, \end{array}\right.$$

where $\beta_{0}>0,\beta_{2}>0$ represent the transmission rate before media alert. The term $\frac{\beta_{1}I_{s}}{m+I_{s}}$, $\frac{\beta_{3}V}{m+V}$ measures the reduction of the virus transmission rate when infection is reported in the media. Because the media coverage cannot prevent the disease from spreading completely, we have $\beta_{0}>\beta_{1}$, $\beta_{2}>\beta_{3}$. The half-saturation constant m>0 reflects the impact of media coverage on the contact transmission. Here, the Holling II type functional response ($\frac{I_{s}}{m+I_{s}}$ and $\frac{V}{m+V}$) is adopted to describe the nature of saturate transmission or psychological effects [25,35,36,37]. The detailed biological meanings of variables and parameters in systems (1) and (2) are listed in Table 1.

The values of most parameters in Table 1 are derived from references [12,15,27]. In order to adapt to the new models, this article has made appropriate modifications to the values of parameters $d_{1}$ and *d* within a reasonable range based on [15].

### 2.2. Methods

(i) The next generation matrix method is used to obtain the basic reproduction number.
(ii) The Descartess rule of signs is used to determine the existence of a positive equilibrium.
(iii) The eigenvalue method, Routh-Hurwitz criteria and LaSalle’s invariance principle are used to prove the stability of two equilibriums.
(iv) The Pontryagin’s maximum principle is used to derive the formula for the optimal solution of System (2).
(v) The Adams-type predictor corrector method and MATLAB software are used for the numerical simulations.

## 3. Results

### 3.1. Qualitative Analysis Results for System (1)

To be biologically meaningful, it is important to prove that the solutions of system (1) with any nonnegative initial data are positive and bounded.

To prove our result, we list the following lemma, which is from [38].

**Lemma** **1.** *Assume that g(t)∈C[a,b] and $D^{\alpha }g(t)\in C\left[a,b\right]$, then we have*

*(i)*  *If $D^{\alpha }g\left(t\right)\geq 0$, for ∀t∈(a,b), then g(t) is non-decreasing for each t∈[a,b].*
*(ii)*  *If $D^{\alpha }g\left(t\right)\leq 0$, for ∀t∈(a,b), then g(t) is non-increasing for each t∈[a,b].*

Denote $X\left(t\right)=(S\left(t\right),I_{s}\left(t\right),I_{a}\left(t\right),V\left(t\right))$ as the solution of system (1) with any positive initial value; then, we have the following result.

**Theorem** **1.** *System (1) always exists a solution X(t) for any positive initial value, and it remains in $R_{+}^{4}$. In addition, the closed set* Γ *is positively invariant for system (1), where*

$$\Gamma =\left\{X\left(t\right)\in R_{+}^{4}:0\leq S+I_{s}+I_{a}\leq \frac{\Lambda }{d},\;0\leq V\leq \left(\frac{hd_{1}}{d}+k\right)\frac{\Lambda }{\varphi }\right\}.$$

The proof of Theorem 1 requires the use of Theorem 3.4 in [39]. The details of the proof is in Appendix A.

Theorem 1 indicates that Γ is positively invariant with respect to system (1). Thus, in the next of this section, we only need to consider the dynamics of system (1) within Γ.

The epidemiological definition of the basic reproduction number is the average number of secondary cases produced by one infected individual introduced into a population of susceptible individuals. By using the next generation matrix method [40], the basic reproduction number of system (1) is derived as follows

(3) $$\begin{array}{ccc} R_{0}=\rho \left(FV^{-1}\right) & = & \frac{p\beta_{0}}{d_{1}}S_{0}+\frac{\left(1-p\right)\beta_{0}\eta }{d}S_{0}+\frac{\beta_{2}}{\varphi }\left[ph+k\left(1-p\right)\right]S_{0}\\ \;\; & ≐ & R_{0s}+R_{0a}+R_{0V}, \end{array}$$

where ρ(*) denotes the spectral radius of a matrix *, and

$$F=\left(\begin{array}{ccc} \;p\beta_{0}S_{0} & p\beta_{0}\eta S_{0} & p\beta_{2}S_{0}\\ \;\left(1-p\right)\beta_{0}S_{0} & \left(1-p\right)\beta_{0}\eta S_{0} & \left(1-p\right)\beta_{2}S_{0}\\ \;0 & 0 & 0 \end{array}\right),\;V=\left(\begin{array}{ccc} d_{1} & 0 & 0\\ \;0 & d & 0\\ \;-hd_{1} & -kd & \varphi \end{array}\right).$$

**Remark** **1.** *In Equation (3), $R_{0s}$, $R_{0a}$, $R_{0V}$ represent the basic reproduction number for the transmission of the symptomatic population, asymptomatic population, and environmental pollutant, respectively.*

In order to obtain the equilibriums and let the right side of system (1) equal to zero, we can obtain the following algebraic equation

(4) $$\left\{\begin{array}{c} \;\Lambda -dS-\left(\beta_{0}-\frac{\beta_{1}I_{s}}{m+I_{s}}\right)S\left(I_{s}+\eta I_{a}\right)-\left(\beta_{2}-\frac{\beta_{3}V}{m+V}\right)SV=0,\\ \;p\left[\left(\beta_{0}-\frac{\beta_{1}I_{s}}{m+I_{s}}\right)S\left(I_{s}+\eta I_{a}\right)+\left(\beta_{2}-\frac{\beta_{3}V}{m+V}\right)SV\right]-d_{1}I_{s}=0,\\ \;\left(1-p\right)\left[\left(\beta_{0}-\frac{\beta_{1}I_{s}}{m+I_{s}}\right)S\left(I_{s}+\eta I_{a}\right)+\left(\beta_{2}-\frac{\beta_{3}V}{m+V}\right)SV\right]-dI_{a}=0,\\ \;hd_{1}I_{s}+kdI_{a}-\varphi V=0. \end{array}\right.$$

A simple calculation shows that in Equation (4) there always exists a trivial solution $E_{0}=\left(\frac{\Lambda }{d},0,0,0\right)$. Denote the positive solution of Equation (4) as $E^{*}=\left(S^{*},I_{s}^{*},I_{a}^{*},V^{*}\right)$; then, we have

(5) $$\begin{array}{c} I_{a}^{*}=\frac{\left(1-p\right)d_{1}I_{s}^{*}}{pd},\;V^{*}=\frac{hd_{1}I_{s}^{*}+kdI_{a}^{*}}{\varphi },\\ S^{*}=\frac{d_{1}I_{s}^{*}}{p\left[\left(\beta_{0}-\frac{\beta_{1}I_{s}^{*}}{m+I_{s}^{*}}\right)\left(I_{s}^{*}+\eta I_{a}^{*}\right)+\left(\beta_{2}-\frac{\beta_{3}V^{*}}{m+V^{*}}\right)V^{*}\right]}.\; \end{array}$$

Substituting Equation (5) into the first equation of the Equation (4), we know that $I_{s}^{*}$ is the non-negative root of the following equation

(6) $$a_{3}I_{s}^{3}+a_{2}I_{s}^{2}+a_{1}I_{s}+a_{0}=0,$$

where

$$\begin{array}{ccc} a_{3} & = & \;-\frac{d_{1}^{2}}{p}\left[hp+k(1-p)\right]\left[\frac{p}{d_{1}}+\frac{\eta (1-p)}{d}\right]\left(\beta_{0}-\beta_{1}\right)-\frac{d_{1}^{2}}{\varphi p}{\left[hp+k(1-p)\right]}^{2}\left(\beta_{2}-\beta_{3}\right)\\ \; & = & -\frac{d_{1}^{2}\upsilon_{1}\upsilon_{2}}{p}\left(\beta_{0}-\beta_{1}\right)-\frac{d_{1}^{2}\upsilon_{1}^{2}}{\varphi p}\left(\beta_{2}-\beta_{3}\right),\\ \;a_{2} & = & \frac{d_{1}}{\varphi }{\left[(hp+k(1-p)\right]}^{2}\left(\beta_{2}-\beta_{3}\right)\left(\Lambda -\frac{d_{1}m}{p}\right)-d_{1}\left[hp+k(1-p)\right]\left(d+\beta_{2}m\right)\\ \; & & +d_{1}\left[hp+k(1-p)\right]\left[\frac{p}{d_{1}}+\frac{\eta (1-p)}{d}\right]\left[\Lambda \left(\beta_{0}-\beta_{1}\right)-\frac{\beta_{0}d_{1}m}{p}\right]\\ \; & = & \frac{d_{1}\upsilon_{1}^{2}}{\varphi }\left(\beta_{2}-\beta_{3}\right)\left(\Lambda -\frac{d_{1}m}{p}\right)-d_{1}\upsilon_{1}\left(d+\beta_{2}m\right)+d_{1}\upsilon_{1}\upsilon_{2}\left[\Lambda \left(\beta_{0}-\beta_{1}\right)-\frac{\beta_{0}d_{1}m}{p}\right],\\ \;a_{1} & = & mpd\varphi \left(R_{0}-1\right)+\Lambda \upsilon_{1}^{2}\frac{d_{1}m}{\varphi }\left(\beta_{2}-\beta_{3}\right)+\beta_{0}m\Lambda d_{1}\upsilon_{1}\upsilon_{2}-\frac{m^{2}\varphi d_{1}}{S_{0}}R_{0}-dd_{1}m\upsilon_{1}\\ \; & & -\Lambda mp\varphi \upsilon_{2}\beta_{1},\\ \;a_{0} & = & p\varphi dm^{2}\left[\frac{p\beta_{0}S_{0}}{d_{1}}+\frac{\eta \left(1-p\right)\beta_{0}S_{0}}{d}+\frac{\beta_{2}S_{0}}{\varphi }\upsilon_{1}-1\right]\\ \; & = & p\varphi dm^{2}\left(R_{0}-1\right), \end{array}$$

and

$$\upsilon_{1}=\left[hp+k(1-p)\right],\;\;\upsilon_{2}=\frac{p}{d_{1}}+\frac{\eta (1-p)}{d}.$$

According to Descartes’s rule of signs [41], we obtain the relation between the signs of the roots and the coefficients $a_{0}$, $a_{1}$, $a_{2}$ and $a_{3}$, which are listed in Table 2.

When $R_{0}>1$, it is easy to obtain $a_{0}>0$, $a_{3}<0$. According to the result in Table 2, we know that Equation (6) has exactly one positive solution, $I^{*}$. Thus, the following result is derived.

**Theorem** **2.**

*(i)*  *In system (1), there always exists a disease-free equilibrium*
*$E_{0}=\left(\frac{\Lambda }{d},0,0,0\right)$.*
*(ii)*  If $R_{0}>1$, then in system (1) there exists a unique endemic equilibrium $E^{*}=\left(S^{*},I_{s}^{*},I_{a}^{*},V^{*}\right)$.

Next, we will discuss the local and global stability of the disease-free equilibrium $E_{0}$.

**Theorem** **3.** *If $R_{0}<1$, then the disease-free equilibrium $E_{0}$ is locally and asymptotically stable for system (1).*

The proof of this theorem is in the Appendix B.

**Theorem** **4.** *If $R_{0}<1$, then the disease-free equilibrium $E_{0}$ is globally and asymptotically stable for system (1).*

**Proof of Theorem** **4.** Consider the following Lyapunov function

$$L=I_{s}\left(t\right)+I_{a}\left(t\right)+\frac{\beta_{2}S_{0}}{\varphi }V\left(t\right),$$

and the derivative of *L* along the solution of system (1) is

$$\begin{array}{ccc} D^{\alpha }{L|}_{\left(1\right)} & = & p\left[\left(\beta_{0}-\frac{\beta_{1}I_{s}}{m+I_{s}}\right)S\left(I_{s}+\eta I_{a}\right)+\left(\beta_{2}-\frac{\beta_{3}V}{m+V}\right)SV\right]-d_{1}I_{s}\;\\ & & +\left(1-p\right)\left[\left(\beta_{0}-\frac{\beta_{1}I_{s}}{m+I_{s}}\right)S\left(I_{s}+\eta I_{a}\right)+\left(\beta_{2}-\frac{\beta_{3}V}{m+V}\right)SV\right]-dI_{a}\;\\ & & +\frac{\beta_{2}S_{0}}{\varphi }\left(hd_{1}I_{s}+kdI_{a}-\varphi V\right)\;\\ & \leq & p\left[\beta_{0}S\left(I_{s}+\eta I_{a}\right)+\beta_{2}SV\right]-d_{1}I_{s}+\left(1-p\right)\left[\beta_{0}S\left(I_{s}+\eta I_{a}\right)+\beta_{2}SV\right]-dI_{a}\;\\ & & +\frac{\beta_{2}S_{0}}{\varphi }hd_{1}I_{s}+\frac{\beta_{2}S_{0}}{\varphi }kdI_{a}-\beta_{2}S_{0}V\;\\ & \leq & \frac{d_{1}I_{s}}{p}\left(\frac{p\beta_{0}S_{0}}{d_{1}}+\frac{\beta_{2}hpS_{0}}{\varphi }-1\right)+\frac{dI_{a}}{1-p}\left[\frac{\left(1-p\right)\beta_{0}\eta S_{0}}{d}+\frac{\left(1-p\right)\beta_{2}kS_{0}}{\varphi }-1\right]\;\\ & < & \frac{d_{1}I_{s}}{p}\left(R_{0}-1\right)+\frac{dI_{a}}{1-p}\left(R_{0}-1\right).\; \end{array}$$

If $R_{0}<1$, we can obtain $D^{\alpha }{V|}_{\left(1\right)}<0$. In addition, $D^{\alpha }{V|}_{\left(1\right)}=0$ if and only if $I_{s}=0$, $I_{a}=0$. The maximum invariant set of system (1) on the set $\left\{\left(S,I_{s},I_{a},V\right)\in \Gamma :D^{\alpha }L{|}_{\left(1\right)}=0\right\}$ is the singleton $\left\{E_{0}\right\}$. According to the LaSalle’s invariance principle, we know that $E_{0}$ is global asymptotically stable if $R_{0}<1$. This completes the proof of the theorem. □

For the endemic equilibrium $E^{*}$ of system (1), we have the following result.

**Theorem** **5.** If $R_{0}$ > 1, then the endemic equilibrium $E^{*}$ exists within *Γ*, and

*(i)*  When α=1, the endemic equilibrium $E^{*}$ is locally asymptotically stable, provided that
$$\begin{array}{c} \kappa_{i}>0,i=0,1,2,3\;and\;\kappa_{3}\kappa_{2}\kappa_{1}>\kappa_{1}^{2}+\kappa_{0}\kappa_{3}^{2}. \end{array}$$
*(ii)*  When α∈(0,1), the above conditions are sufficient but not necessary for the local asymptotic stability of the endemic equilibrium $E^{*}$. In fact, $E^{*}$ is still locally asymptotically stable if all eigenvalues $\lambda_{i}$ of Equation (A4) satisfy
$$\left|\arg(\lambda_{i})\right|>\frac{\alpha \pi }{2}.$$

The proof of this theorem can be derived according to the Routh-Hurwitz criteria [42] and Lemma 4 in [43], and the detailed proof is in Appendix C.

### 3.2. Examples and Numerical Simulation Results for System (1)

In this subsection, we will establish some examples and perform some numerical simulations to verify the results obtained in the previous subsection. In addition, the sensitive analysis of some parameters is also taken for system (1). In particular, the effect of media coverage is discussed. In this paper, we use an Adams-type predictor-corrector method and MATLAB software to solve fractional order differential and integral equations.

**Example** **1.** *Fix the following parameter values: Λ=1700, $\beta_{0}=5.4646e-10$, $\beta_{1}=4.4646e-10$, $\beta_{2}=3.8024e-11$, $\beta_{3}=2.8024e-11$, m=30, d=0.004060, η=0.7001, p=0.7899, $d_{1}=0.006040$, k=299.99, h=10 and φ=0.03264. In this case, we obtain $R_{0}=0.0287<1$.*

**Figure 1:**
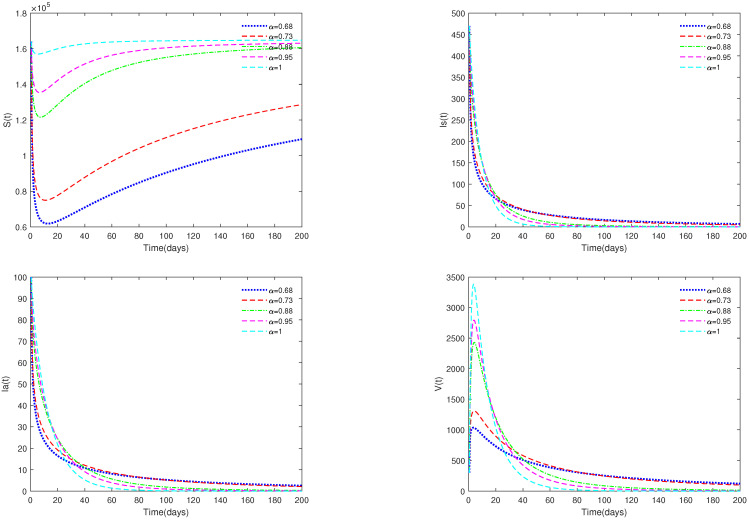
Time series of system (1) for different values of α. Here, $R_{0}=0.0287<1$.

**Figure 2:**
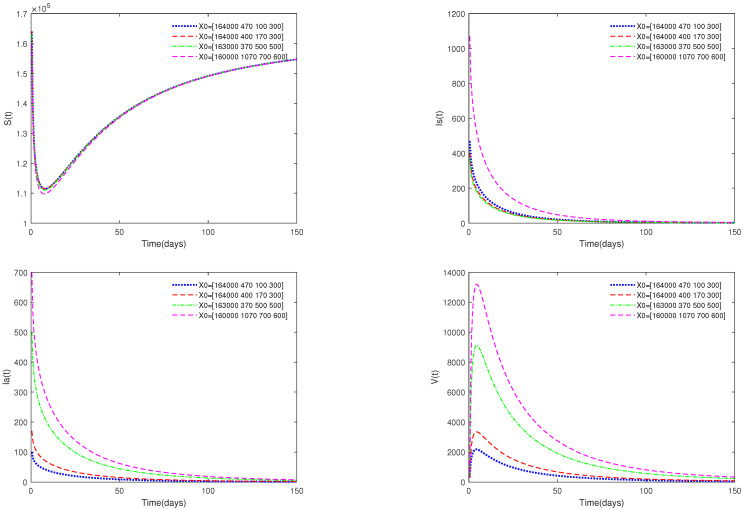
Time series of system (1) for different initial values. Here, $R_{0}=0.0287<1$.

*(i)*  *In Figure 1, the initial value is $X_{0}$ = [164,000, 470, 100, 300], and α have different values (α=0.68,0.73,0.88,0.95,1). Figure 1 shows that if $R_{0}=0.0287<1$, then the disease-free equilibrium $E_{0}$ is always asymptotically stable for for all α∈[0.68,1].*
*(ii)*  *In Figure 2, the value of α is fixed to 0.85, and different initial values are taken. $X_{0}$ = [164,000, 470, 100, 300], [164,000, 400, 170, 300], [163,000, 370, 500, 500], [160,000, 1070, 700, 600]. Figure 2 indicates that different initial values do not affect the stability of the disease-free equilibrium $E_{0}$ of system (1).*

**Example** **2.** *Fix the following parameter values: Λ=304, $\beta_{0}=5.4646e-7$, $\beta_{1}=4.4646e-7$, $\beta_{2}=3.8024e-8$, $\beta_{3}=2.8024e-8$, m=30, d=0.004060, η=0.7001, p=0.7899, $d_{1}=0.006040$, k=299.99, h=10 and φ=0.03264. In this case, we get $R_{0}=13.0203>1$.*

**Figure 3:**
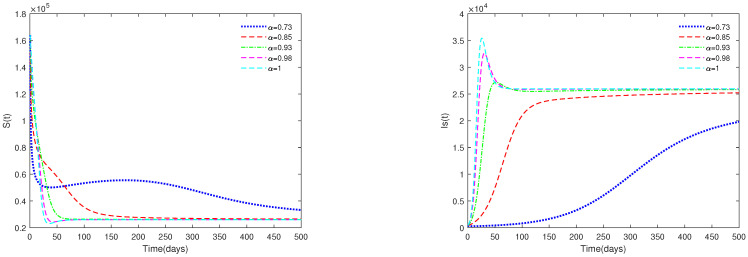

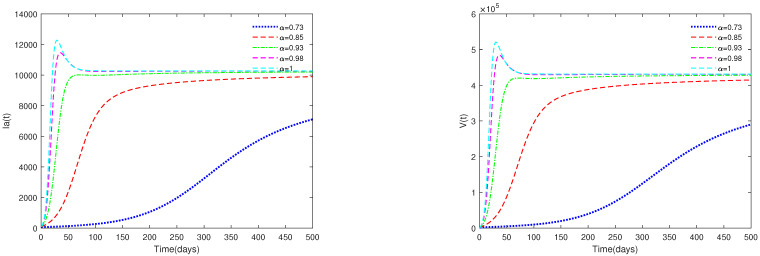
Time series of system (1) for different values of α. Here, $R_{0}=13.0203>1$.

**Figure 4:**
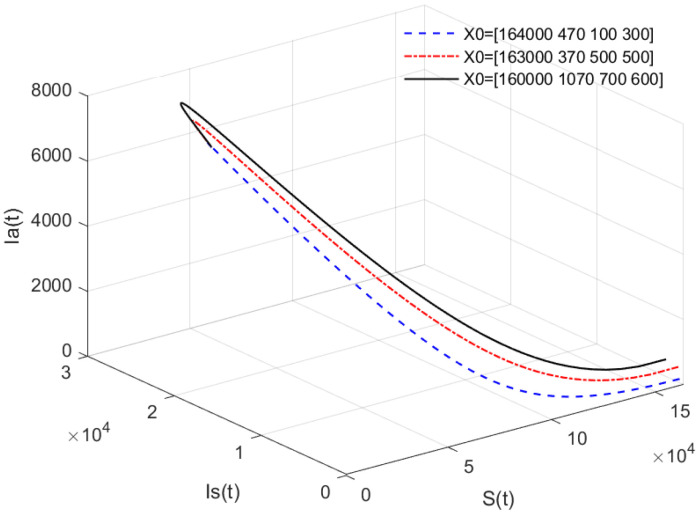
Phase portrait of S(t), $I_{s}\left(t\right)$, and $I_{a}\left(t\right)$ for different initial values. Here, $R_{0}=13.0203>1$.

*(i)*  In Figure 3, the initial value is fixed to $X_{0}$ = [164,000, 470, 100, 300], and α have different values (α=0.73,0.85,0.93,0.98,1). Figure 3 shows that if $R_{0}=13.0203>1$, then the endemic equilibrium $E^{*}$ is always asymptotically stable for all α∈[0.73,1].
*(ii)*  In Figure 4, the value of α is fixed to α=0.95, and different initial values are taken as $X_{0}$ = [164,000, 470, 100, 300], [163,000, 370, 500, 500], [160,000, 1070, 700, 600]. Figure 4 indicates that different initial values do not affect the stability of the endemic equilibrium $E^{*}$ of system (1).

**Example** **3.** *For the following parameter values: Λ=304, $\beta_{0}=5.4646e-7$, $\beta_{1}=4.4646e-7$, $\beta_{2}=3.8024e-8$, $\beta_{3}=2.8024e-8$, m=30, η=0.7001, p=0.7899, $d_{1}=0.006040$, k=299.99, h=10 and φ=0.03264.*

**Figure 5:**
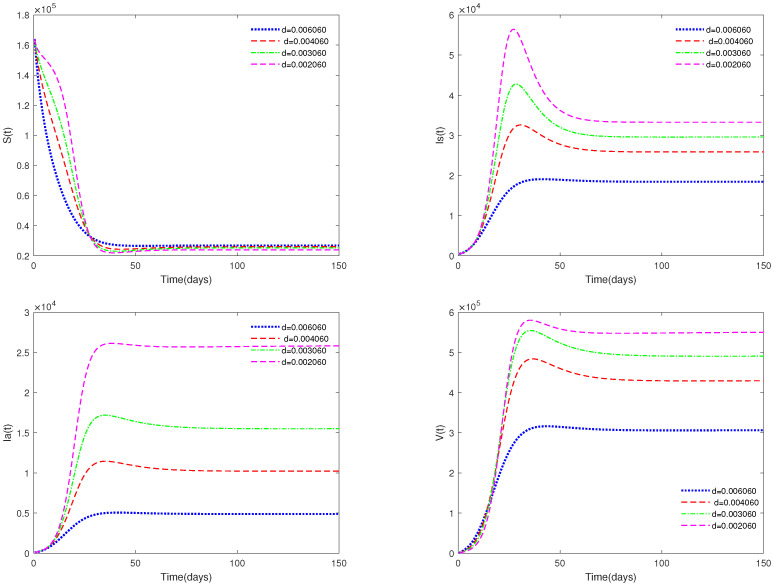
Time series of system (1) for different values of *d*.

**Figure 6:**
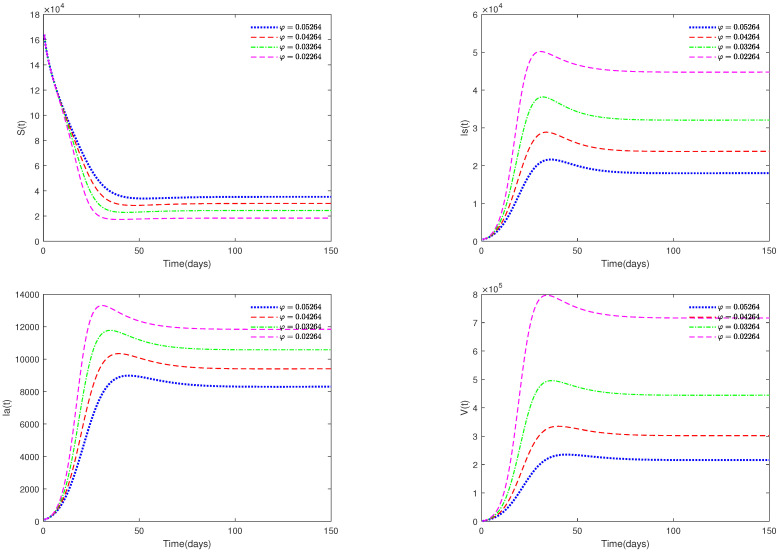
Time series of system (1) for different values of φ.

*(i)*  *In Figure 5, the value of α is fixed to 0.9, and different values for d (d = 0.0060608, 0.004060, 0.003060, 0.002060) are taken, in order to analyze the sensitivity of the mortality parameter d on system (1).*
*(ii)*  *In Figure 6, the value α is fixed to 0.98, and different values for φ (φ = 0.05264, 0.04264, 0.03264, 0.02264) are taken, in order to analyze the sensitivity of parameter φ on system (1).*

**Example** **4.** *Fix the following parameter values: Λ=304, $\beta_{0}=5.4646e-7$, $\beta_{2}=3.8024e-8$, m=30, d=0.004060, η=0.7001, p=0.7899, $d_{1}=0.006040$, k=299.99, h=10 and φ=0.03264.*

**Figure 7:**
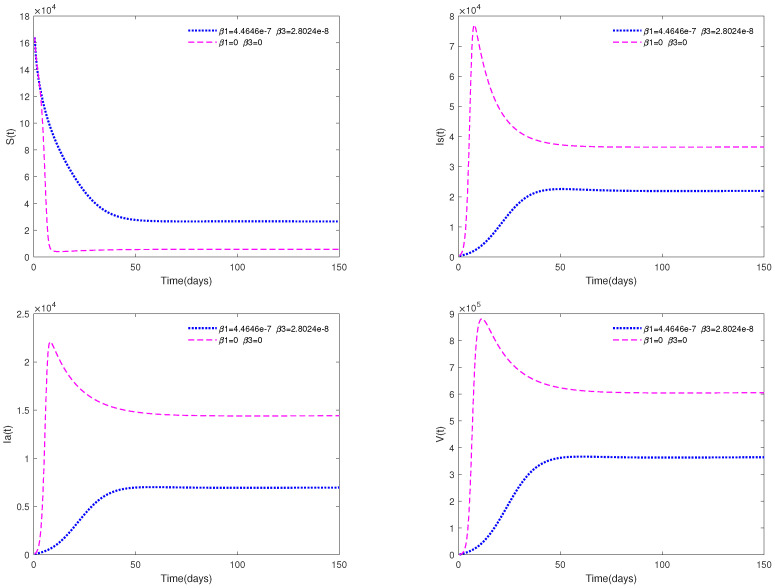
Time series of system (1) with media coverage (in blue) or without media coverage (in purple).

Figure 7 shows the impact of media coverage on system (1). From this figure, we can observe that the infected population (both $I_{s}$ and $I_{a}$) will tend to be a higher level if there is no media coverage ($\beta_{1}=0$, $\beta_{3}=0$); while they will tend to be a lower level if there is media coverage ($\beta_{1}=4.4646e-7$, $\beta_{3}=2.8024e-8$).

The results of the numerical simulation are summarized, as follows.

**Remark** **2.**

*(i)*  *Figure 1 and Figure 2 show that if $R_{0}<1$, then the disease-free equilibrium $E_{0}$ is always stable. If the basic reproduction number $R_{0}<1$, that is, the number of healthy pigs infected by a diseased pig during its average disease period does not exceed 1, then the disease will eventually disappear, and this result is consistent with reality. The value of α can affect the speed towards the equilibrium. The initial values will not affect the stability, which is in line with Theorems 1 and 4.*
*(ii)*  Figure 3 and Figure 4 indicate that if $R_{0}>1$, then the disease-free equilibrium $E_{0}$ is unstable and the endemic equilibrium exists. If the basic reproduction number $R_{0}>1$, that is, if the number of healthy pigs infected by a diseased pig during its average disease period is more than 1, then the disease will break out in this region and become an endemic. The value of α will affect the speed towards the endemic equilibrium $E^{*}$. The initial values will not affect the stability, which is in accordance with Theorems 1 and 2.
*(iii)*  *Figure 5 and Figure 6 show the sensitivity analysis for parameters d and φ. Through observation, it can be observed that the mortality rate d of pigs and the clearance rate φ of viruses have a significant impact on system (1). Therefore, it is reasonable for us to consider specific control measures in system (2) as removing diseased pigs and strengthening the disinfection and sterilization of pig breeding environments.*
*(iv)*  *Figure 7 shows the significant impact of media coverage on system (1). That is to say, if the media can timely convey the news to local pig farmers in the early stage of an ASF outbreak in a region, then it can greatly reduce the number of infected pigs and help to prevent further spread.*

### 3.3. Qualitative Analysis Results for System (2)

In this subsection, we will analyze the fractional optimal control of system (2). Similar to the approach in Section 3.1, it is easy to prove that system (2) with any positive initial value has a unique positive solution that remains within Γ.

Next, we will use the Hamiltonian function and Pontryagin’s Maximum Principle [44] to describe the optimal control problem.

The objective function is defined as

(7) $$J\left(u_{1},u_{2}\right)=\int_{0}^{T}\left[A_{1}I_{s}\left(t\right)+A_{2}V\left(t\right)+\frac{1}{2}B_{1}u_{1}^{2}\left(t\right)+\frac{1}{2}B_{2}u_{2}^{2}\left(t\right)\right]dt,$$

where *T* is the final time, and the parameters $A_{1}$, $A_{2}$ are positive constants to keep a balance in the size of $I_{s}\left(t\right)$ and V(t); $B_{1}$, $B_{2}$ are positive weight parameters which are associated with the control measures $u_{1}\left(t\right)$ and $u_{2}\left(t\right)$. In system (2), the control measures $u_{1}\left(t\right)$ and $u_{2}\left(t\right)$, represent the clearance rate of the suspected diseased population and the elimination rate of bacteria in the environment by using alkaline disinfectant at a given time *t*, respectively. The goal is to minimize the populations which are already infected and the contaminants in the environment. Thus, we need to find the optimal solution $u_{1}^{*}$ and $u_{2}^{*}$ that satisfy

$$J\left(u_{1}^{*},u_{2}^{*}\right)=\min\left\{J\left(u_{1},u_{2}\right),\left(u_{1},u_{2}\right)\in U\right\},$$

where

$$U=\{\vec{u}=\left(u_{1},\;u_{2}\right)|u_{i}\left(t\right)\;\mathrm{is}\;\mathrm{Lebesgue}\;\mathrm{measurable},\;0\leq u_{i}\left(t\right)\leq 1,\;t\in \left[0,\;T\right],\;i=1,\;2\}.$$

Define the Lagrangian and Hamiltonian function, as follows.

Lagrangian function

(8) $$G\left(I_{s},V,u_{1},u_{2}\right)=A_{1}I_{s}+A_{2}V+\frac{1}{2}B_{1}u_{1}^{2}\left(t\right)+\frac{1}{2}B_{2}u_{2}^{2}\left(t\right),$$

Hamiltonian function

$$\begin{array}{ccc} \;H & = & A_{1}I_{s}+A_{2}V+\frac{1}{2}B_{1}u_{1}^{2}\left(t\right)+\frac{1}{2}B_{2}u_{2}^{2}\left(t\right)\\ \; & + & \vartheta_{1}\left[\Lambda -dS-\left(\beta_{0}-\frac{\beta_{1}I_{s}}{m+I_{s}}\right)S\left(I_{s}+\eta I_{a}\right)-\left(\beta_{2}-\frac{\beta_{3}V}{m+V}\right)SV\right]\\ \; & + & \vartheta_{2}\left[p\left(\beta_{0}-\frac{\beta_{1}I_{s}}{m+I_{s}}\right)S\left(I_{s}+\eta I_{a}\right)+p\left(\beta_{2}-\frac{\beta_{3}V}{m+V}\right)SV-d_{1}I_{s}-u_{1}\left(t\right)I_{s}\right]\\ \; & + & \vartheta_{3}\left[\left(1-p\right)\left(\beta_{0}-\frac{\beta_{1}I_{s}}{m+I_{s}}\right)S\left(I_{s}+\eta I_{a}\right)+\left(1-p\right)\left(\beta_{2}-\frac{\beta_{3}V}{m+V}\right)SV-dI_{a}\right]\\ \; & + & \vartheta_{4}\left[hd_{1}I_{s}+kdI_{a}-\varphi V-u_{2}\left(t\right)V\right], \end{array}$$

where $\vartheta_{i}\left(t\right)$, i=1,2,3,4, are the adjoint variables.

**Theorem** **6.** Given the optimal control $\left(u_{1}^{*}\left(t\right),u_{2}^{*}\left(t\right)\right)$ and the corresponding solution $(S^{*}\left(t\right),I_{s}^{*}\left(t\right),$$I_{a}^{*}\left(t\right),V^{*}\left(t\right))$ of system (2), there exists adjoint variables $\vartheta_{i}\left(t\right),i=1,2,3,4$ satisfying the following equations.

(9) $$\left\{\begin{array}{ccc} D^{\alpha }\vartheta_{1}\left(t\right) & = & \vartheta_{1}\left[d+\left(\beta_{0}-\frac{\beta_{1}I_{s}^{*}}{m+I_{s}^{*}}\right)\left(I_{s}^{*}+\eta I_{a}^{*}\right)+\left(\beta_{2}-\frac{\beta_{3}V^{*}}{m+V^{*}}\right)V^{*}\right]\;\\ \; & - & \vartheta_{2}p\left[\left(\beta_{0}-\frac{\beta_{1}I_{s}^{*}}{m+I_{s}^{*}}\right)\left(I_{s}^{*}+\eta I_{a}^{*}\right)+\left(\beta_{2}-\frac{\beta_{3}V^{*}}{m+V^{*}}\right)V^{*}\right]\;\\ \; & - & \vartheta_{3}\left(1-p\right)\left[\left(\beta_{0}-\frac{\beta_{1}I_{s}^{*}}{m+I_{s}^{*}}\right)\left(I_{s}^{*}+\eta I_{a}^{*}\right)+\left(\beta_{2}-\frac{\beta_{3}V^{*}}{m+V^{*}}\right)V^{*}\right],\;\\ D^{\alpha }\vartheta_{2}\left(t\right) & = & -A_{1}-\vartheta_{1}\left[\frac{\beta_{1}m}{{\left(m+I_{s}^{*}\right)}^{2}}\left(I_{s}^{*}+\eta I_{a}^{*}\right)S^{*}-\left(\beta_{0}-\frac{\beta_{1}I_{s}^{*}}{m+I_{s}^{*}}\right)S^{*}\right]\;\\ \; & - & \vartheta_{2}p\left[\left(\beta_{0}-\frac{\beta_{1}I_{s}^{*}}{m+I_{s}^{*}}\right)S^{*}-\frac{\beta_{1}m}{{\left(m+I_{s}^{*}\right)}^{2}}\left(I_{s}^{*}+\eta I_{a}^{*}\right)S^{*}\right]+\vartheta_{2}d_{1}+\vartheta_{2}u_{1}^{*}\;\\ \; & - & \vartheta_{3}\left(1-p\right)\left[\left(\beta_{0}-\frac{\beta_{1}I_{s}^{*}}{m+I_{s}^{*}}\right)S^{*}-\frac{\beta_{1}m}{{\left(m+I_{s}^{*}\right)}^{2}}\left(I_{s}^{*}+\eta I_{a}^{*}\right)S^{*}\right]-\vartheta_{4}hd_{1},\;\\ D^{\alpha }\vartheta_{3}\left(t\right) & = & \vartheta_{1}\left(\beta_{0}-\frac{\beta_{1}I_{s}^{*}}{m+I_{s}^{*}}\right)S^{*}\eta -\vartheta_{2}p\left(\beta_{0}-\frac{\beta_{1}I_{s}^{*}}{m+I_{s}^{*}}\right)S^{*}\eta \;\\ \; & - & \vartheta_{3}\left(1-p\right)\left(\beta_{0}-\frac{\beta_{1}I_{s}^{*}}{m+I_{s}^{*}}\right)S^{*}\eta +\vartheta_{3}d-\vartheta_{4}kd,\;\\ D^{\alpha }\vartheta_{4}\left(t\right) & = & -A_{2}-\vartheta_{1}\left[\frac{\beta_{3}m}{{\left(m+V^{*}\right)}^{2}}S^{*}V^{*}-\left(\beta_{2}-\frac{\beta_{3}V^{*}}{m+V^{*}}\right)S^{*}\right]+\vartheta_{4}\varphi +\vartheta_{4}u_{2}^{*}\;\\ \; & - & \vartheta_{2}p\left[\left(\beta_{2}-\frac{\beta_{3}V^{*}}{m+V^{*}}\right)S^{*}-\frac{\beta_{3}m}{{\left(m+V^{*}\right)}^{2}}S^{*}V^{*}\right]\;\\ \; & - & \vartheta_{3}\left(1-p\right)\left[\left(\beta_{2}-\frac{\beta_{3}V^{*}}{m+V^{*}}\right)S^{*}-\frac{\beta_{3}m}{{\left(m+V^{*}\right)}^{2}}S^{*}V^{*}\right], \end{array}\right.$$

*with the transversal conditions*

$$\vartheta_{i}\left(T\right)=0,\;i=1,2,3,4.$$

Furthermore, for t∈[0,T], the formula of optimal solution $u_{1}^{*}\left(t\right),u_{2}^{*}\left(t\right)$ are given by

$$u_{1}^{*}\left(t\right)=\min\left\{\max\left\{\frac{\vartheta_{2}I_{s}^{*}}{B_{1}},\;0\right\},\;1\right\},$$

$$u_{2}^{*}\left(t\right)=\min\left\{\max\left\{\frac{\vartheta_{4}V^{*}}{B_{2}},\;0\right\},\;1\right\}.$$

The proof of this theorem is in Appendix D.

### 3.4. Examples and Numerical Simulation Results for System (2)

**Example** **5.** *Fix the following parameter values: Λ=304, $\beta_{0}=5.4646e-7$, $\beta_{1}=4.4646e-7$, $\beta_{2}=3.8024e-8$, $\beta_{3}=2.8024e-8$, m=30, d=0.004060, η=0.7001, p=0.7899, $d_{1}=0.006040$, k=299.99, h=10, φ=0.03264, $A_{1}=0.2$, $A_{2}=0.8$, $B_{1}=0.5$ and $B_{2}=0.5$. In this case, we obtain $R_{0}=13.0203>1$.*

**Figure 8:**
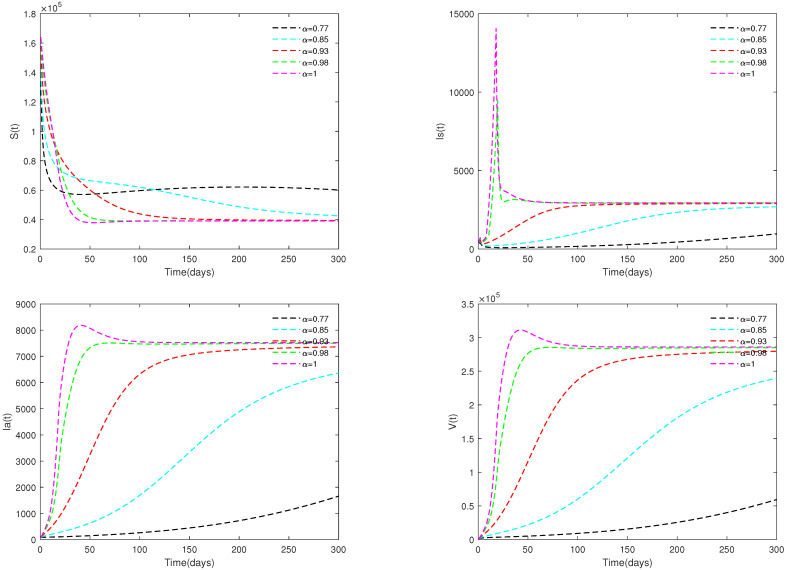
Optimal solutions for system (2) with different values of α. Here, $R_{0}=13.0203>1$.

*Fix the initial value as $X_{0}$ = [164,000, 470, 100, 300], and different α values (α=0.77,0.85,0.93, 0.98 and 1) are taken. Figure 8 shows that the value of parameter α affects the speed towards the stable state.*

**Example** **6.** *For the following parameter values: Λ=304, $\beta_{0}=5.4646e-7$, $\beta_{2}=3.8024e-8$, m=30, d=0.004060, η=0.7001, p=0.7899, $d_{1}=0.006040$, k=299.99, h=10, φ=0.03264 and initial value $X_{0}$ = [164,000, 470, 100, 300].*

**Figure 9:**
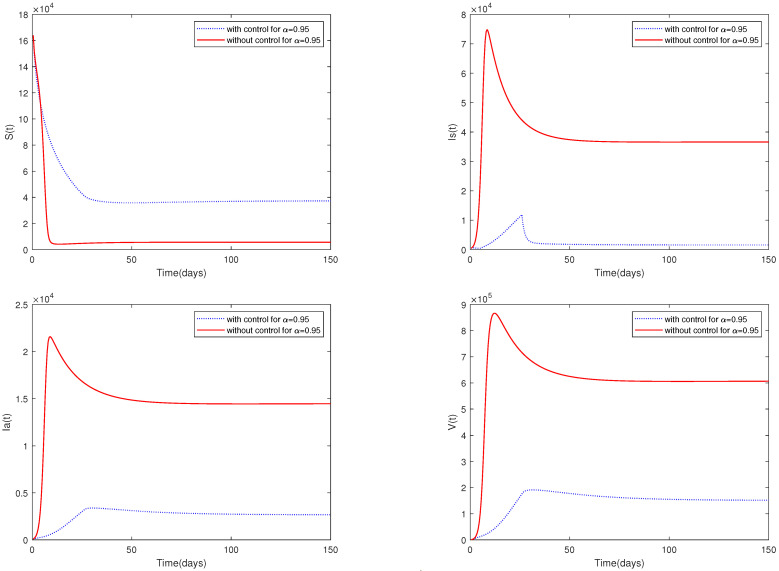
Optimal solutions for system (2) with control measures or without control measures.

Figure 9 shows the dynamic behavior of system (2) with control measures and media coverage ($\beta_{1}=3.34646e-7$, $\beta_{3}=3.7024e-8$, $u_{1}=u_{1}^{*}\left(t\right)$, $u_{2}=u_{2}^{*}\left(t\right)$) or without control measures and media coverage ($\beta_{1}=0$, $\beta_{3}=0$, $u_{1}=0$, $u_{2}=0$).

**Example** **7.** *Fix the following parameter values: Λ=304, $\beta_{0}=5.4646e-7$, $\beta_{1}=4.4646e-7$, $\beta_{2}=3.8024e-8$, $\beta_{3}=2.8024e-8$, m=30, d=0.004060, η=0.7001, p=0.7899, $d_{1}=0.006040$, k=299.99, h=10, φ=0.03264, $A_{1}=0.2$, $A_{2}=0.8$, $B_{1}=0.5$ and $B_{2}=0.5$.*

**Figure 10:**
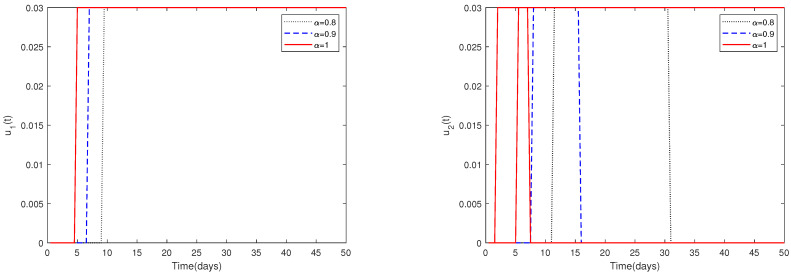
The optimal solutions of $u_{1}^{*}\left(t\right)$ and $u_{2}^{*}\left(t\right)$ for system (2) with different values of α. Here, the upper limit of $u_{1}\left(t\right)$, $u_{2}\left(t\right)$ is relatively small (realistically reasonable).

**Figure 11:**
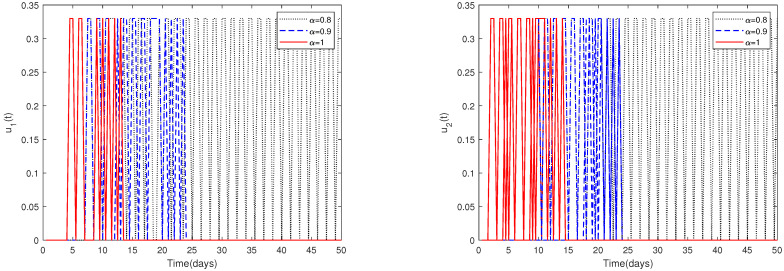
The optimal solutions of $u_{1}^{*}\left(t\right)$ and $u_{2}^{*}\left(t\right)$ for system (2) with different values of α. Here, the upper limit of $u_{1}\left(t\right)$, $u_{2}\left(t\right)$ is relatively big (realistically unreasonable).

*(i)*  In Figure 10, the initial value is fixed to $X_{0}$ = [164,000, 470, 100, 300]. For different values of α, this figure demonstrates the optimal solution of $u_{1}^{*}\left(t\right)$ and $u_{2}^{*}\left(t\right)$ when the upper limit of $u_{1}\left(t\right)$, $u_{2}\left(t\right)$ is relatively small (realistically reasonable).
*(ii)*  In Figure 11, the initial value is fixed to $X_{0}$ = [164,000, 470, 100, 300]. For different values of α, this figure shows the optimal solution of $u_{1}^{*}\left(t\right)$ and $u_{2}^{*}\left(t\right)$ when the upper limit of $u_{1}\left(t\right)$, $u_{2}\left(t\right)$ is relatively larger (realistically unreasonable).

The numerical simulation results for system (2) are summarized as follows.

**Remark** **3.**

*(i)*  *When $R_{0}=13.0203>1$, Figure 8 shows that for all α∈[0.77,1], the corresponding optimal solution tend to be stable with different speeds. This indicates that under the values of Example 3, ASF will outbreak in a certain region, and it will gradually become an endemic.*
*(ii)*  *A comparison between Figure 7 and Figure 9 shows that media coverage combined with control measures can suppress the spread of ASF more effectively. That is to say, if pig farmers take timely measures to eliminate suspected infected pigs and disinfect the environment of pig farms on a large scale after receiving media reports of the outbreak of ASF in the local area, they can greatly reduce the infection and help to prevent the spread of the epidemic.*
*(iii)*  *Since the magnitudes change dramatically for different parameters, choosing a suitable upper limit of $u_{1}\left(t\right)$, $u_{2}\left(t\right)$ is important.*
Figure 10 shows that if the upper limit of $u_{1}\left(t\right)$, $u_{2}\left(t\right)$ is relatively small, then the optimal control solutions $u_{1}^{*}\left(t\right)$ and $u_{2}^{*}\left(t\right)$ can be suitably solved. However, if the upper limit of $u_{1}\left(t\right)$, $u_{2}\left(t\right)$ is relatively big, then the optimal control solutions $u_{1}^{*}$ and $u_{2}^{*}$ cannot be solved suitably, as shown in Figure 11. In fact, obvious and chaotic oscillations occur.

## 4. Discussion

In this paper, two fractional order African swine fever models are established to study the effect of media coverage. For the first model, no control measures are taken, and the existence and stability of equilibriums are analyzed. For the second model, the optimal solution is derived by using Pontryagin’s maximal principle.

The main results of the qualitative analysis and numerical simulation for system (1) are as follows.

- There always exists a unique positive solution for any positive initial value, and the set Γ is positively invariant for this system. This conclusion is essential from a biological perspective.
- The basic reproduction number $R_{0}$ is obtained.
- The sufficient conditions for the existence and stability of the disease-free equilibrium $E_{0}$ and endemic equilibrium $E^{*}$ are derived.
- From Figure 1, Figure 2, Figure 3 and Figure 4, it can be observed that the initial value is not crucial and it does not affect the stability. This means that the initial value of susceptible pigs and diseased pigs is not a key factor. However, the value of α is important, and it will affect the speed towards a stable state. This result indicates that the fractional order system is different from its corresponding integer order system.
- Figure 5 and Figure 6 show that both parameters *d* and φ are sensitive. In fact, *d* and φ have a significant effect on the basic reproduction number $R_{0}$. In practice, we can reduce the value of $R_{0}$ by increasing the mortality rate of diseased pigs or increasing disinfection measures in pig houses, thereby achieving the goal of preventing the continued spread of the disease.
- Figure 7 shows that media coverage is a very useful measure to control the disease.

The main results of qualitative analysis and numerical simulation for system (2) are as follows.

- The formula of the optimal control solution $u_{1}^{*}\left(t\right)$ and $u_{2}^{*}\left(t\right)$ is obtained by using the Pontryagin’s maximum principle.
- Figure 9 indicates that media coverage combined with control measures (such as disinfection and sterilization) can suppress the spread of the disease more effectively.
- Figure 10 and Figure 11 indicate that choosing a suitable upper limit of $u_{1}\left(t\right)$, $u_{2}\left(t\right)$ is important, otherwise, oscillation behavior will occur.

## 5. Conclusions

As there are currently no effective drugs to treat ASF, preventive measures and biosecurity play an important role in preventing the outbreak of the epidemic. Research has demonstrated that the earlier control measures are taken, the easier it is to reduce the harm caused by the disease [13]. In this article, the effect of media coverage is investigated for the outbreak or elimination of ASF. Considering the memory advantage of fractional order systems, we introduced media coverage and optimal control into the mathematical model to simulate the development process of the disease. Through numerical simulations, we find that when pig farmers receive news from the media about the outbreak of ASF, timely measures such as clearing suspected diseased pigs and disinfecting pig farms can significantly reduce the number of infected pigs and reduce economic losses.

This article considers the dissemination role of media coverage on an epidemic. In fact, there are more professional tools available to monitor the spread of animal diseases, such as the Geographic Information System (GIS) software used in [45] and GIS and the Remote Sensing used in [46]. In addition, stochastic differential equations are also useful to describe the transmission of diseases. Thus, stochastic effects will be taken into consideration to construct more reasonable and realistic models in the future work.

## Figures and Tables

**Table 1:** The biological meanings for the variables and parameters in systems (1) and (2).

Variables	Description			
S(t)	Density of the susceptible population			
$I_{s}\left(t\right)$	Density of the symptomatic infectious population			
$I_{a}\left(t\right)$	Density of the asymptomatic infectious population			
V(t)	Density of ASFV in the environment			
Parameters	Description	Value	Units	Refs
Λ	The recruitment rate of population	670	${\mathrm{day}}^{-1}$	[12]
$\beta_{0}$	ASFV transmission rate with direct contact of infectious population	$5.46\times 10^{-10}$	${\mathrm{day}}^{-1}$	[15]
$\beta_{2}$	Virus transmission rate of contaminated pig products and materials	$3.80\times 10^{-11}$	${\mathrm{day}}^{-1}$	[15]
*m*	The half-saturation constant	30	−−	[27]
η	Reduced rate by asymptomatic population	0.7001	${\mathrm{day}}^{-1}$	[15]
*p*	The proportion of symptomatic infectious population	0.7899	−−	[15]
$d_{1}$	Natural and disease related death rate of population	0.006040	${\mathrm{day}}^{-1}$	−−
*d*	Natural death rate of population	0.004060	${\mathrm{day}}^{-1}$	−−
*h*	The release rate of virus from symptomatic infectious population	10.0575	${\mathrm{day}}^{-1}$	[15]
*k*	The release rate of virus from asymptomatic infectious population	299.6462	${\mathrm{day}}^{-1}$	[15]
φ	Virus clearance rate	0.3264	${\mathrm{day}}^{-1}$	[15]
$u_{1}\left(t\right)$	Measures to eliminate suspected disease population	[0, 1]	${\mathrm{day}}^{-1}$	−−
$u_{2}\left(t\right)$	Measures for disinfection and sterilization with disinfectant	[0, 1]	${\mathrm{day}}^{-1}$	−−

**Table 2:** Descartes Sign Rule for Equation (6).

Number and Sign of Roots	$a_{3}$	$a_{2}$	$a_{1}$	$a_{0}$
3 negative	+	+	+	+
3 positive	+	−	+	−
1 positive	+	−	−	−
1 positive	+	+	−	−
1 positive	+	+	+	−
1 positive	−	+	+	+
2 positive	+	−	+	+
2 positive	+	−	−	+

## Data Availability

The data presented in this study are available in the manuscript.

## Appendix A. Proof of Theorem 1

**Proof of Theorem** **1.** Firstly, we will prove that the solution of system (1) with any positive initial values is always non-negative and bounded. From system (1), we easily obtain

$$\begin{array}{ccc} \;D^{\alpha }{S|}_{S=0} & = & \Lambda \geq 0,\\ \;D^{\alpha }I_{s}{|}_{I_{s}=0} & = & p\left[\beta_{0}S\eta I_{a}+\left(\beta_{2}-\frac{\beta_{3}V}{m+V}\right)SV\right]\geq 0,\\ \;D^{\alpha }I_{a}{|}_{I_{a}=0} & = & \left(1-p\right)\left[\left(\beta_{0}-\frac{\beta_{1}I_{s}}{m+I_{s}}\right)SI_{s}+\left(\beta_{2}-\frac{\beta_{3}V}{m+V}\right)SV\right]\geq 0,\\ \;D^{\alpha }{V|}_{V=0} & = & hd_{1}I_{s}+kdI_{a}\geq 0. \end{array}$$

According to Lemma 1 and the above inequalities, we have X(t)≥0 for any t≥0. Adding the first three equations of system (1), we obtain

$$D^{\alpha }N\left(t\right)=\Lambda -dS\left(t\right)-d_{1}I_{s}\left(t\right)-dI_{a}\left(t\right)\leq \Lambda -dN\left(t\right),$$

which implies that

$$N\left(t\right)\leq \left[-\frac{\Lambda }{d}+N\left(0\right)\right]E_{\alpha }\left(-dt^{\alpha }\right)+\frac{\Lambda }{d}.$$

Since $E_{\alpha }\left(-dt^{\alpha }\right)\geq 0$ for any t≥0, then we have

$$N\left(t\right)\leq \frac{\Lambda }{d},\;\forall \;t\geq 0,$$

provided that $N\left(0\right)\leq \frac{\Lambda }{d}$. Similarly, from the fourth equation of system (1), we obtain

$$D^{\alpha }V\left(t\right)\leq hd_{1}\frac{\Lambda }{d}+k\Lambda -\varphi V\left(t\right),$$

which implies that

$$V\left(t\right)\leq \left[-\left(\frac{hd_{1}}{d}+k\right)\frac{\Lambda }{\varphi }+V\left(0\right)\right]E_{\alpha }\left(-\varphi t^{\alpha }\right)+\left(\frac{hd_{1}}{d}+k\right)\frac{\Lambda }{\varphi }.$$

Since $E_{\alpha }\left(-\varphi t^{\alpha }\right)\geq 0$ for any t≥0, then we obtain

$$V\left(t\right)\leq \left(\frac{hd_{1}}{d}+k\right)\frac{\Lambda }{\varphi },\;\forall \;t\geq 0,$$

provided that $V\left(0\right)\leq \left(\frac{hd_{1}}{d}+k\right)\frac{\Lambda }{\varphi }$. Secondly, we will prove that system (1) with any positive initial value has a unique solution. Denote the right side of system (1) as vector function $f(t,\vec{x}\left(t\right))$, and

$$\vec{x}\left(t\right)=\left(\begin{array}{c} x_{1}\left(t\right)\\ x_{2}\left(t\right)\\ x_{3}\left(t\right)\\ x_{4}\left(t\right) \end{array}\right),\xi =\left(\begin{array}{c} \Lambda \\ 0\\ 0\\ 0 \end{array}\right),A_{1}=\left(\begin{array}{cccc} -d & 0 & 0 & 0\\ 0 & -d_{1} & 0 & 0\\ 0 & 0 & -d & 0\\ 0 & hd_{1} & kd & -\varphi \end{array}\right),$$

$$\begin{array}{c} A_{2}=\left(\begin{array}{cccc} 0 & -\beta_{0} & -\beta_{0}\eta & -\beta_{2}\\ 0 & p\beta_{0} & p\beta_{0}\eta & p\beta_{2}\\ 0 & \left(1-p\right)\beta_{0} & \left(1-p\right)\beta_{0}\eta & \left(1-p\right)\beta_{2}\\ 0 & 0 & 0 & 0 \end{array}\right), \end{array}$$

$$A_{3}=\left(\begin{array}{cccc} 0 & 1 & \eta & 0\\ 0 & -p & -p\eta & 0\\ 0 & -(1-p) & -(1-p)\eta & 0\\ 0 & 0 & 0 & 0 \end{array}\right),A_{4}=\left(\begin{array}{cccc} 0 & 0 & 0 & 1\\ 0 & 0 & 0 & -p\\ 0 & 0 & 0 & -(1-p)\\ 0 & 0 & 0 & 0 \end{array}\right),$$

where $x_{1}\left(t\right)=S\left(t\right)$, $x_{2}\left(t\right)=I_{s}\left(t\right)$, $x_{3}\left(t\right)=I_{a}\left(t\right)$, $x_{4}\left(t\right)=V\left(t\right)$, $x_{1}\left(0\right)=S\left(0\right)$, $x_{2}\left(0\right)=I_{s}\left(0\right)$, $x_{3}\left(0\right)=I_{a}\left(0\right)$, $x_{4}\left(0\right)=V\left(0\right)$. Then, system (1) can be written as

$$\begin{array}{ccc} D^{\alpha }\vec{x}\left(t\right) & = & A_{1}\vec{x}\left(t\right)+x_{1}\left(t\right)A_{2}\vec{x}\left(t\right)+\frac{x_{1}\left(t\right)\beta_{1}x_{2}\left(t\right)}{m+x_{2}\left(t\right)}A_{3}\vec{x}\left(t\right)+\frac{x_{1}\left(t\right)\beta_{3}x_{4}\left(t\right)}{m+x_{4}\left(t\right)}A_{4}\vec{x}\left(t\right)+\xi \\ & ≐ & f(t,\vec{x}\left(t\right)), \end{array}$$

Obviously, the corresponding conditions (i)–(iii) of Theorem 3.1 in [39] are satisfied. Furthermore, we have

$$\begin{array}{ccc} \;∥f(t,\vec{x}\left(t\right))∥ & = & ∥A_{1}\vec{x}\left(t\right)+x_{1}\left(t\right)A_{2}\vec{x}\left(t\right)+\frac{x_{1}\left(t\right)\beta_{1}x_{2}\left(t\right)}{m+x_{2}\left(t\right)}A_{3}\vec{x}\left(t\right)+\frac{x_{1}\left(t\right)\beta_{3}x_{4}\left(t\right)}{m+x_{4}\left(t\right)}A_{4}\vec{x}\left(t\right)+\xi ∥\\ \; & \leq & (∥A_{1}∥+∥x_{1}\left(t\right)A_{2}∥+∥x_{1}\left(t\right)∥\cdot ∥\frac{\beta_{1}x_{2}\left(t\right)}{m+x_{2}\left(t\right)}∥\cdot ∥A_{3}∥\\ \; & & +∥x_{1}\left(t\right)∥\cdot ∥\frac{\beta_{3}x_{4}\left(t\right)}{m+x_{4}\left(t\right)}∥\cdot ∥A_{4}∥)∥\vec{x}\left(t\right)∥+∥\xi ∥\\ \; & \leq & \left(∥A_{1}∥+\frac{\Lambda }{d}∥A_{2}∥+\frac{\Lambda }{d}∥A_{3}∥+\frac{\Lambda }{d}∥A_{4}∥\right)∥\vec{x}\left(t\right)∥+∥\xi ∥\\ \; & ≐ & \mu ∥\vec{x}\left(t\right)∥+\theta . \end{array}$$

Thus, the fourth condition of Theorem 3.1 in [39] is also satisfied for system (1). According to that theorem, we know that system (1) has a unique positive solution for any positive initial value. This completes the proof. □

## Appendix B. Proof of Theorem 3

**Proof of Theorem** **3.** The Jacobian matrix of system (1), evaluated at the disease-free equilibrium $E_{0}$, is given by

$$J\left(E_{0}\right)=\left(\begin{array}{cccc} \;-d & -\beta_{0}S_{0} & -\beta_{0}\eta S_{0} & -\beta_{2}S_{0}\\ \;0 & p\beta_{0}S_{0}-d_{1} & p\beta_{0}\eta S_{0} & p\beta_{2}S_{0}\\ \;0 & \left(1-p\right)\beta_{0}S_{0} & \left(1-p\right)\beta_{0}\eta S_{0}-d & \left(1-p\right)\beta_{2}S_{0}\\ \;0 & hd_{1} & kd & -\varphi \end{array}\right),$$

and the corresponding characteristic equation will be

(A1) $$\left|\begin{array}{cccc} \;\lambda +d & \beta_{0}S_{0} & \beta_{0}\eta S_{0} & \beta_{2}S_{0}\\ \;0 & \lambda -p\beta_{0}S_{0}+d_{1} & -p\beta_{0}\eta S_{0} & -p\beta_{2}S_{0}\\ \;0 & -\left(1-p\right)\beta_{0}S_{0} & \lambda -\left(1-p\right)\beta_{0}\eta S_{0}+d & -\left(1-p\right)\beta_{2}S_{0}\\ \;0 & -hd_{1} & -kd & \lambda +\varphi \end{array}\right|=0.$$

It is easy to observe that $\lambda_{1}=-d$ is a negative eigenvalue, and the remaining eigenvalues satisfy the following equation

(A2) $$\begin{array}{ccc} \;\left(\lambda +d\right)\left(\lambda +d_{1}\right)\left(\lambda +\varphi \right) & = & \left(1-p\right)\beta_{0}\eta S_{0}\left(\lambda +d_{1}\right)\left(\lambda +\varphi \right)+kd\left(1-p\right)\beta_{2}S_{0}\left(\lambda +d_{1}\right)\\ \;\; & \; & +phd_{1}\beta_{2}S_{0}\left(\lambda +d\right)+p\beta_{0}\eta S_{0}\left(\lambda +d\right)\left(\lambda +\varphi \right). \end{array}$$

If the roots of Equation (A2) satisfy Re(λ)≥0, then we have

$$\begin{array}{ccc} \;1 & = & \left|\frac{\left(1-p\right)\beta_{0}\eta S_{0}}{\lambda +d}+\frac{kd\left(1-p\right)\beta_{2}S_{0}}{\left(\lambda +d)(\lambda +\varphi \right)}+\frac{phd_{1}\beta_{2}S_{0}}{\left(\lambda +d_{1}\right)\left(\lambda +\varphi \right)}+\frac{p\beta_{0}\eta S_{0}}{\lambda +d_{1}}\right|\\ \; & \leq & \left|\frac{\left(1-p\right)\beta_{0}\eta S_{0}}{d}|+|\frac{kd\left(1-p\right)\beta_{2}S_{0}}{d\varphi }|+|\frac{phd_{1}\beta_{2}S_{0}}{d_{1}\varphi }|+|\frac{p\beta_{0}\eta S_{0}}{d_{1}}\right|\\ \; & \leq & \frac{\left(1-p\right)\beta_{0}\eta S_{0}}{d}+\frac{k\left(1-p\right)\beta_{2}S_{0}}{\varphi }+\frac{ph\beta_{2}S_{0}}{\varphi }+\frac{p\beta_{0}S_{0}}{d_{1}}\\ \; & = & \frac{p\beta_{0}}{d_{1}}S_{0}+\frac{\left(1-p\right)\beta_{0}\eta }{d}S_{0}+\frac{\beta_{2}}{\varphi }\left(ph+k\left(1-p\right)\right)S_{0}\\ \; & = & R_{0}. \end{array}$$

Since $R_{0}<1$, the above inequality is obviously invalid, that is, we will obtain Re(λ)<0. Thus, all roots of Equation (A1) have negative real parts, which indicates that the disease-free equilibrium $E_{0}$ is locally asymptotically stable for system (1). □

## Appendix C. Proof of Theorem 5

**Proof of Theorem** **5.** The Jacobian matrix of system (1) evaluated at the endemic equilibrium $E^{*}$ is given by

(A3) $$J\left(E^{*}\right)=\left(\begin{array}{cccc} \omega_{1} & \omega_{2} & \omega_{3} & \omega_{4}\\ \omega_{5} & \omega_{6} & \omega_{7} & \omega_{8}\\ \omega_{9} & \omega_{10} & \omega_{11} & \omega_{12}\\ 0 & \omega_{13} & \omega_{14} & \omega_{15} \end{array}\right),$$

where

$$\begin{array}{ccc} \;\omega_{1}=-\frac{\Lambda }{S^{*}}, & & \omega_{2}=\left[\frac{\beta_{1}m}{{\left(m+I_{s}^{*}\right)}^{2}}\left(I_{s}^{*}+\eta I_{a}^{*}\right)-\left(\beta_{0}-\frac{\beta_{1}I_{s}^{*}}{m+I_{s}^{*}}\right)\right]S^{*},\\ \;\omega_{3}=-\left(\beta_{0}-\frac{\beta_{1}I_{s}^{*}}{m+I_{s}^{*}}\right)\eta S^{*}, & & \omega_{4}=\left[\frac{\beta_{3}mV^{*}}{{\left(m+V^{*}\right)}^{2}}-\left(\beta_{2}-\frac{\beta_{3}V^{*}}{m+V^{*}}\right)\right]S^{*},\\ \;\omega_{5}=\frac{d_{1}I_{s}^{*}}{S^{*}}, & & \omega_{6}=-p\left[\frac{\beta_{1}m}{{\left(m+I_{s}^{*}\right)}^{2}}\left(I_{s}^{*}+\eta I_{a}^{*}\right)-\left(\beta_{0}-\frac{\beta_{1}I_{s}^{*}}{m+I_{s}^{*}}\right)\right]S^{*}-d_{1},\\ \;\omega_{7}=p\left[\beta_{0}-\frac{\beta_{1}I_{s}^{*}}{m+I_{s}^{*}}\right]\eta S^{*}, & & \omega_{8}=-p\left[\frac{\beta_{3}mV^{*}}{{\left(m+V^{*}\right)}^{2}}-\left(\beta_{2}-\frac{\beta_{3}V^{*}}{m+V^{*}}\right)\right]S^{*},\\ \;\omega_{9}=\frac{dI_{a}^{*}}{S^{*}}, & & \omega_{10}=-\left(1-p\right)\left[\frac{\beta_{1}m}{{\left(m+I_{s}^{*}\right)}^{2}}\left(I_{s}^{*}+\eta I_{a}^{*}\right)-\left(\beta_{0}-\frac{\beta_{1}I_{s}^{*}}{m+I_{s}^{*}}\right)\right]S^{*},\\ \;\omega_{11}=\left(1-p\right)\left[\beta_{0}-\frac{\beta_{1}I_{s}^{*}}{m+I_{s}^{*}}\right]\eta S^{*}, & & \omega_{12}=-\left(1-p\right)\left[\frac{\beta_{3}mV^{*}}{{\left(m+V^{*}\right)}^{2}}-\left(\beta_{2}-\frac{\beta_{3}V^{*}}{m+V^{*}}\right)\right]S^{*},\\ \;\omega_{13}=hd_{1}, & & \omega_{14}=kd,\;\omega_{15}=-\varphi . \end{array}$$

The corresponding characteristic equations will be

(A4) $$\lambda^{4}+\kappa_{3}\lambda^{3}+\kappa_{2}\lambda^{2}+\kappa_{1}\lambda +\kappa_{0}=0,$$

where

$$\begin{array}{ccc} \;\kappa_{0} & = & \omega_{1}\left(\omega_{6}\omega_{11}\omega_{15}+\omega_{6}\omega_{12}\omega_{14}-\omega_{7}\omega_{10}\omega_{15}+\omega_{7}\omega_{12}\omega_{13}+\omega_{8}\omega_{10}\omega_{14}-\omega_{8}\omega_{11}\omega_{13}\right)\\ \; & & +\omega_{2}\left(\omega_{5}\omega_{12}\omega_{14}-\omega_{5}\omega_{11}\omega_{15}+\omega_{7}\omega_{9}\omega_{15}-\omega_{8}\omega_{9}\omega_{14}\right)\\ \; & & +\omega_{3}\left(\omega_{5}\omega_{10}\omega_{15}-\omega_{5}\omega_{12}\omega_{13}-\omega_{6}\omega_{9}\omega_{15}+\omega_{8}\omega_{9}\omega_{13}\right)\\ \; & & +\omega_{4}\left(\omega_{5}\omega_{11}\omega_{13}-\omega_{5}\omega_{10}\omega_{14}+\omega_{6}\omega_{9}\omega_{14}-\omega_{7}\omega_{9}\omega_{13}\right),\;\\ \;\kappa_{1} & = & \omega_{1}\omega_{7}\omega_{10}-\omega_{1}\omega_{6}\omega_{11}+\omega_{2}\omega_{5}\omega_{11}-\omega_{2}\omega_{7}\omega_{9}-\omega_{3}\omega_{5}\omega_{10}+\omega_{3}\omega_{6}\omega_{9}-\omega_{1}\omega_{6}\omega_{15}\\ \; & & +\omega_{1}\omega_{8}\omega_{13}+\omega_{2}\omega_{5}\omega_{15}-\omega_{4}\omega_{5}\omega_{13}-\omega_{1}\omega_{11}\omega_{15}+\omega_{1}\omega_{12}\omega_{14}+\omega_{3}\omega_{9}\omega_{15}-\omega_{4}\omega_{9}\omega_{14}\\ \; & & -\omega_{6}\omega_{11}\omega_{15}+\omega_{6}\omega_{12}\omega_{14}+\omega_{7}\omega_{10}\omega_{15}-\omega_{7}\omega_{12}\omega_{13}{-}_{8}\omega_{10}\omega_{14}+\omega_{8}\omega_{11}\omega_{13},\;\\ \;\kappa_{2} & = & \omega_{1}\omega_{6}-\omega_{2}\omega_{5}+\omega_{1}\omega_{11}-\omega_{3}\omega_{9}+\omega_{1}\omega_{15}+\omega_{6}\omega_{11}-\omega_{7}\omega_{10}+\omega_{6}\omega_{5}\\ & & -\omega_{8}\omega_{13}+\omega_{11}\omega_{15}-\omega_{12}\omega_{14},\;\\ \;\kappa_{3} & = & -(\omega_{1}+\omega_{6}+\omega_{11}+\omega_{15}). \end{array}$$

According to the Routh–Hurwitz criteria [42] and Lemma 4 in [43], this theorem is derived. □

## Appendix D. Proof of Theorem 6

**Proof of Theorem** **6.** The adjoint equations and transversal conditions of optimal control problems can be easily obtained from Pontriagin’s maximum principle, as follows. Adjoint equations

(A5) $$\left\{\begin{array}{ccccc} D^{\alpha }\vartheta_{1}\left(t\right) & = & -\frac{\partial H}{\partial S} & = & \vartheta_{1}\left[d+\left(\beta_{0}-\frac{\beta_{1}I_{s}^{*}}{m+I_{s}^{*}}\right)\left(I_{s}^{*}+\eta I_{a}^{*}\right)+\left(\beta_{2}-\frac{\beta_{3}V^{*}}{m+V^{*}}\right)V^{*}\right]\;\\ \; & \; & & - & \vartheta_{2}p\left[\left(\beta_{0}-\frac{\beta_{1}I_{s}^{*}}{m+I_{s}^{*}}\right)\left(I_{s}^{*}+\eta I_{a}^{*}\right)+\left(\beta_{2}-\frac{\beta_{3}V^{*}}{m+V^{*}}\right)V^{*}\right]\;\\ \; & \; & & - & \vartheta_{3}\left(1-p\right)\left[\left(\beta_{0}-\frac{\beta_{1}I_{s}^{*}}{m+I_{s}^{*}}\right)\left(I_{s}^{*}+\eta I_{a}^{*}\right)+\left(\beta_{2}-\frac{\beta_{3}V^{*}}{m+V^{*}}\right)V^{*}\right],\;\\ D^{\alpha }\vartheta_{2}\left(t\right) & = & -\frac{\partial H}{\partial I_{s}} & = & -A_{1}-\vartheta_{1}\left[\frac{\beta_{1}m}{{\left(m+I_{s}^{*}\right)}^{2}}\left(I_{s}^{*}+\eta I_{a}^{*}\right)S^{*}-\left(\beta_{0}-\frac{\beta_{1}I_{s}^{*}}{m+I_{s}^{*}}\right)S^{*}\right]\;\\ \; & \; & & - & \vartheta_{2}p\left[\left(\beta_{0}-\frac{\beta_{1}I_{s}^{*}}{m+I_{s}^{*}}\right)S^{*}-\frac{\beta_{1}m}{{\left(m+I_{s}^{*}\right)}^{2}}\left(I_{s}^{*}+\eta I_{a}^{*}\right)S^{*}\right]+\vartheta_{2}d_{1}+\vartheta_{2}u_{1}^{*}\;\\ \; & \; & & - & \vartheta_{3}\left(1-p\right)\left[\left(\beta_{0}-\frac{\beta_{1}I_{s}^{*}}{m+I_{s}^{*}}\right)S^{*}-\frac{\beta_{1}m}{{\left(m+I_{s}^{*}\right)}^{2}}\left(I_{s}^{*}+\eta I_{a}^{*}\right)S^{*}\right]-\vartheta_{4}hd_{1},\;\\ D^{\alpha }\vartheta_{3}\left(t\right) & = & -\frac{\partial H}{\partial I_{a}} & = & \vartheta_{1}\left(\beta_{0}-\frac{\beta_{1}I_{s}^{*}}{m+I_{s}^{*}}\right)S^{*}\eta -\vartheta_{2}p\left(\beta_{0}-\frac{\beta_{1}I_{s}^{*}}{m+I_{s}^{*}}\right)S^{*}\eta \;\\ \; & \; & & - & \vartheta_{3}\left(1-p\right)\left(\beta_{0}-\frac{\beta_{1}I_{s}^{*}}{m+I_{s}^{*}}\right)S^{*}\eta +\vartheta_{3}d-\vartheta_{4}kd,\;\\ D^{\alpha }\vartheta_{4}\left(t\right) & = & -\frac{\partial H}{\partial V} & = & -A_{2}-\vartheta_{1}\left[\frac{\beta_{3}m}{{\left(m+V^{*}\right)}^{2}}S^{*}V^{*}-\left(\beta_{2}-\frac{\beta_{3}V^{*}}{m+V^{*}}\right)S^{*}\right]+\vartheta_{4}\varphi +\vartheta_{4}u_{2}^{*}\;\\ \; & \; & & - & \vartheta_{2}p\left[\left(\beta_{2}-\frac{\beta_{3}V^{*}}{m+V^{*}}\right)S^{*}-\frac{\beta_{3}m}{{\left(m+V^{*}\right)}^{2}}S^{*}V^{*}\right]\;\\ \; & \; & & - & \vartheta_{3}\left(1-p\right)\left[\left(\beta_{2}-\frac{\beta_{3}V^{*}}{m+V^{*}}\right)S^{*}-\frac{\beta_{3}m}{{\left(m+V^{*}\right)}^{2}}S^{*}V^{*}\right],\; \end{array}\right.$$

with transversal conditions

$$\vartheta_{1}\left(T\right)=0,\;\vartheta_{2}\left(T\right)=0,\;\vartheta_{3}\left(T\right)=0,\;\vartheta_{4}\left(T\right)=0.$$

The optimal control pairs $u_{1}^{*}\left(t\right)$ and $u_{2}^{*}\left(t\right)$ can be derived by the following equations

$$\begin{array}{ccccc} \;\frac{\partial H}{\partial u_{1}} & = & 0⟹B_{1}u_{1}^{*}-\vartheta_{2}I_{s}^{*} & = & 0,\\ \frac{\partial H}{\partial u_{2}} & = & 0⟹B_{2}u_{2}^{*}-\vartheta_{4}V^{*} & = & 0. \end{array}$$

Combining with the definition of optimal control solutions, we get the final formula as follows

$$u_{1}^{*}\left(t\right)=\min\left\{\max\left\{\left(\frac{\vartheta_{2}I_{s}^{*}}{B_{1}}\right),\;0\right\},\;1\right\},$$

$$u_{2}^{*}\left(t\right)=\min\left\{\max\left\{\frac{\vartheta_{4}V^{*}}{B_{2}},\;0\right\},\;1\right\}.$$

This completes the proof. □
